# A novel indicator in epidemic monitoring through a case study of Ebola in West Africa (2014–2016)

**DOI:** 10.1038/s41598-024-62719-3

**Published:** 2024-05-27

**Authors:** Minkyu Kwak, Xiuxiu Sun, Yunju Wi, Kyeongah Nah, Yongkuk Kim, Hongsung Jin

**Affiliations:** 1 Department of Mathematics and Statistics, Chonnam National University, Gwangju, South Korea; 2 Department of Mathematics and Physics, Luoyang Institute of Science and Technology, Henan, China; 3 Busan Center for Medical Mathematics, National Institute of Mathematical Sciences, Busan, South Korea; 4 Department of Mathematics, Kyungpook National University, Daegu, South Korea

**Keywords:** $E/S$ ratio, Cross point (CP), Time-dependent reproduction number, Time-dependent transmission rate, SEIR model, Ebola outbreak, Computational biology and bioinformatics, Diseases, Mathematics and computing

## Abstract

The *E/S* (exposed/susceptible) ratio is analyzed in the SEIR model. The ratio plays a key role in understanding epidemic dynamics during the 2014–2016 Ebola outbreak in Sierra Leone and Guinea. The maximum value of the ratio occurs immediately before or after the time-dependent reproduction number (R_*t*_) equals 1, depending on the initial susceptible population (*S*(0)). It is demonstrated that transmission rate curves corresponding to various incubation periods intersect at a single point referred to as the Cross Point (CP). At this point, the *E/S* ratio reaches an extremum, signifying a critical shift in transmission dynamics and aligning with the time when R_*t*_ approaches 1. By plotting transmission rate curves, *β*(*t*), for any two arbitrary incubation periods and tracking their intersections, we can trace CP over time. CP serves as an indicator of epidemic status, especially when R_*t*_ is close to 1. It provides a practical means of monitoring epidemics without prior knowledge of the incubation period. Through a case study, we estimate the transmission rate and reproduction number, identifying CP and R_*t*_ = 1 while examining the *E/S* ratio across various values of *S*(0).

## Introduction

The time-dependent reproduction number serves as a measure indicating the effectiveness of disease control^1–6^. Estimating the time-dependent reproduction number, ${\text{R}}_{t}$, depends on how the model structure is defined, which epidemiological features it should include, and what kind of data are available^7,8^. A challenge with the assessment of the time-dependent reproduction number with SIR (susceptible–infectious–recovered) type of models is the estimation of the transmission rate, especially when it varies over the course of the epidemic due to the implementation of control policies^3,4,8^. Some approaches assume explicit form of the functions on the several pieces of time intervals which are determined by the timing of control policies and the epidemic situation^9^. Pollicott et al.^10^ estimated the time-dependent transmission rate (*β*(*t*)) of a SIR model by solving a linear ordinary differential equation (ODE) of *β*(*t*) when the removal rate (γ) is a given value. Applying this method, Wang et al.^11^ developed an approach to utilize a machine learning algorithm to estimate the transmission rate with the time-dependent variables reflecting the intensity of non-pharmaceutical policies. Nadler et al.^12^ incorporated these types of time-dependent data through variational data assimilation. Grimm et al.^13^ partitioned the time interval into several shorter interval on each of which they assumed constant transmission rates. They estimated these rates using a machine learning approach involving physics-informed neural networks.

In this study, we introduce a point in the epidemic curve called CP, which has the potential to serve as an indicator signifying that the disease is nearing control. This point can be identified without prior knowledge of the incubation period, making it a useful measure to determine the epidemic status when the time-dependent reproduction number is nearing one. The CP may occur before or after ${\text{R}}_{t}=1$, but it indicates proximity to this critical value. To obtain CP, the parameters of the SEIR system are estimated by solving the inverse problem^10,14–18^. Then, the system is rearranged to construct the time-dependent transmission rate and the time-dependent reproduction number. We prove that for any incubation period, the transmission rate curves pass through CP, where $\frac{{d}}{{{dt}}}\left( {E/S} \right)=0$. The intersection point of the time-dependent transmission rate curves for two arbitrary incubation periods becomes CP. The role of the $E/S$ ratio is investigated in determining which point, between CP and ${\text{R}}_{t}=1$ 1, appears first. As *S*(0) increases, the extreme value of the $E/S$ ratio converges to a constant and CP appears before ${\text{R}}_{t}=1$.

To simulate the process, we utilize data from the Ebola outbreak in Sierra Leone and Guinea. This outbreak began in Guinea in December 2013 and later spread to other West African countries, including Liberia and Sierra Leone, resulting in nearly 30,000 infections from 2014 to 2016^19–22^. In this case study, we estimate the dates of occurrence for CP and ${\text{R}}_{t}=1$ and calculate the time difference between them. The time-dependent reproduction number is reached to one after a few days after CP, with its value being greater than one at the time of CP. Therefore, CP holds the potential to be used as a precautionary indicator signifying that the disease is nearing control.

## Materials and methods

A population ${N}$ is partitioned into compartments labeled *S, E, I,* and *R*, representing susceptible, exposed, infectious, and removed individuals, respectively. The model includes four parameters: time-dependent transmission rate $\left(\beta \left(t\right)\right)$, rate of progression from exposure to infection $\left(\sigma \right)$, removal rate ($\gamma$), and case fatality rate ($f$) ^23^. The SEIR model analyzed in this paper has several assumptions. Firstly, it assumes that the size of the total population remains constant: this assumption corresponds to the net input to the susceptible by births being equal to the net mortality^24^. This simplification is often adopted to focus solely on the dynamics of disease transmission without considering demographic changes. Secondly, the population is assumed to be homogeneous, implying that individuals within each compartment are considered identical in terms of susceptibility, exposure, infectiousness, and recovery^25^. Thirdly the population is assumed to be well-mixed, with individuals having an equal chance of coming into contact with any other individual^25–27^. This assumption facilitates modeling the spread of the disease in a population where interactions are random and frequent. Fourthly, once individuals recover from the infectious stage, they gain immunity and cannot be infected again, at least for some period^28^.

The transmission dynamics are described as follows:

1 $$\left\{ {\begin{array}{*{20}l} {\frac{{dS}}{{dt}} = - {\frac{{\beta \left( t \right)SI}}{N}}} \hfill \\ {\frac{{dE}}{{dt}} = {\frac{{\beta \left( t \right)SI}}{N}} - \sigma E} \hfill \\ {\frac{{dI}}{{dt}} = \sigma E - \gamma I} \hfill \\ {\frac{{dR}}{{dt}} = \left( {1 - f} \right)\gamma I} \hfill \\ {N = S + E + I + R} \hfill \\ \end{array} } \right.$$

### The point of $\frac{{d}}{{{dt}}}\left( {E/S} \right)=0$ and incubation periods

For various incubation periods, the transmission rate curves pass through a single point, as depicted in Fig. 3a,c. This point of intersection is denoted as CP. It is proven that the point where $\frac{{d}}{{{dt}}}\left( {E/S} \right)=0$ coincides with the cross point. CP is independent of the incubation period $(1/\sigma )$, and it can be easily estimated by plotting two transmission rate curves for any two incubation periods.

#### Theorem 1

The transmission rate $(\beta \left(t\right))$ shares a single common point where $\frac{{d}}{{{dt}}}\left( {E/S} \right)=0$. At this point, the value of $\beta \left(t\right)$ is independent of $\upsigma$^29^.

#### ***Proof***

Let $${f}_{1}(t)\equiv \sigma E$$ and $${f}_{2}\left(t\right)\equiv S+E$$.

Then,

$$S=-{\frac{{f}_{1}(t)}{\sigma }}+{f}_{2}\left(t\right)$$

From Eq. (1)

$${\frac{dS}{dt}}=-{\frac{\beta \left(t\right)SI}{N}}$$

$${\frac{dE}{dt}}={\frac{\beta \left(t\right)SI}{N}}-\sigma E$$

one has

$$\beta \left( t \right) = - \frac{{\frac{dS}{{dt}}}}{\frac{SI}{N}} = \frac{{\frac{{f_{1}}^{\prime } (t)}{\sigma } - {f_{2}}^{\prime } (t)}}{{ - \frac{{f_{1} (t)}}{\sigma } + f_{2} \left( t \right)}}\frac{N}{I} = \frac{{f_{1}}^{\prime } (t) - \sigma {f_{2}}^{\prime } (t)}{{ - f_{1} \left( t \right) + \sigma f_{2} \left( t \right)}}\frac{N}{I}$$

and

$$\frac{\partial \beta }{\partial \sigma }=\frac{{f}_{1}\left(t\right){{f}_{2}}^{\prime}(t)-{f}_{2}(t){{f}_{1}}^{\prime}(t)}{{(-{f}_{1}\left(t\right)+\sigma {f}_{2}\left(t\right))}^{2}}\frac{N}{I}=0$$

Thus, $\beta \left(t\right)$ is independent of $\upsigma$ when ${f}_{1}\left(t\right){{f}_{2}}^{\prime}(t)={f}_{2}(t){{f}_{1}}^{\prime}(t)$ or $E{S}^{\prime}={E}^{\prime}S$

*or* $$\frac{{d}}{{{dt}}}\left( {E/S} \right)=0.$$

### The value of ${\text{R}}_{t}$ at CP

To compare the times of $\frac{{d}}{{{dt}}}\left( {E/S} \right)=0$ and ${\text{R}}_{t}=1$, the value of ${\text{R}}_{t}$ is investigated when $\frac{{d}}{{{dt}}}\left( {E/S} \right)=0$. The time-dependent reproduction number can be expressed as^23^,

2 $$\begin{array}{c}{\text{R}}_{t}= \frac{\beta \left(t\right)S}{\gamma N}\end{array}$$

By rearranging the second equation in Eq. (1), the transmission rate can be written as:

3 $$\begin{array}{c}\beta \left(t\right)=\frac{N}{SI}\left(\frac{dE}{dt}+\sigma E\right)\end{array}$$

Then, ${\text{R}}_{t}$ can be written as:

4 $$\begin{array}{c}{\text{R}}_{t}= \frac{SE^{\prime}-ES^{\prime}}{\upgamma I\left(S+E\right)}+\frac{\sigma E}{\upgamma I }\frac{1}{\left(1+E/S\right)}\end{array}$$

Since $S{E}^{\prime}-E{S}^{\prime}=0$ at the point where $\frac{{d}}{{{dt}}}\left( {E/S} \right)=0$, the time-dependent reproduction number at CP can be written as:

5 $$\begin{array}{c}{\text{R}}_{t}= \frac{\sigma E}{\upgamma I }\frac{1}{\left(1+E/S\right)}\end{array}$$

The value of ${\text{R}}_{t}$ at CP can be used to estimate which event occurs first: CP or ${\text{R}}_{t}=1$.

There are 3 cases of ${\text{R}}_{t}$ values at CP:

(i) If $\frac{\sigma E}{\upgamma I }\frac{1}{\left(1+E/S\right)}>1$, then ${\text{R}}_{t}>1$ and CP appears earlier than ${\text{R}}_{t}=1$
(ii) If $\frac{\sigma E}{\upgamma I }\frac{1}{\left(1+E/S\right)}=1$, then CP and ${\text{R}}_{t}=1$ appear at the same time.
(iii) If $\frac{\sigma E}{\upgamma I }\frac{1}{\left(1+E/S\right)}<1$, then ${\text{R}}_{t}<1$ and CP appears later than ${\text{R}}_{t}=1$.

In the SEIR model, $\sigma E$ represents the number of newly infected people entering compartment ${I}$, and $\upgamma I$ represents the number of infected people leaving compartment ${I}$. The ratio of $\frac{\sigma E}{\upgamma I }$ is entirely dependent on the variation in compartment ${I}$. It is always greater than one when $dI/dt>0$, as indicated by Eq. (1). Additionally, the ratio $E/S$ also plays a crucial role in determining the value of ${\text{R}}_{t}$ at $\frac{{d}}{{{dt}}}\left( {E/S} \right)=0.$ At this point, $E/S$ reaches a maximum while $\frac{1}{\left(1+E/S\right)}$ attains a minimum value. When *S*(0) is sufficiently large so that ${S}\gg {E}$, CP always appears earlier than ${\text{R}}_{t}=1$. Therefore, CP can be a precautionary indicator that ${\text{R}}_{t}=1$ is imminent. Assuming that the initial susceptible population $S(0)$ is very small, the effect of $E/S$ cannot be ignored in Eq. (5), and CP can appear after ${\text{R}}_{t}=1$. Although the appearance time of CP and ${\text{R}}_{t}=1$ is dependent on the value of $E/S$, they are very close for usual cases between $dE/dt=0$ and $dI/dt=0$, where $\frac{\sigma E}{\upgamma I }\approx 1$ and $\frac{1}{\left(1+E/S\right)}\approx 1$. Therefore, CP can be an alternative indicator to ${\text{R}}_{t}=1$, suggesting that the epidemic is almost under control when $\frac{{d}}{{{dt}}}\left( {E/S} \right)=0$.

## Case study

In this study, we utilized the data of cumulative cases and deaths from the Ebola outbreak in Guinea and Sierra Leone, sourced from the World Health Organization^30^. The dataset covered a period of 2 years: for Guinea, we included 262 data points reported from March 25, 2014, to March 26, 2016, and for Sierra Leone, 196 data points reported from May 27, 2014, to October 30, 2015.

### Cumulative case and death data

The time-dependent transmission rate and reproduction number in the SEIR model are estimated using only two regression functions for the cumulative cases and deaths data. Two equations, $\frac{dC}{dt}$ and $\frac{dD}{dt}$, are added to the SEIR system in Eq. (6). Here, C represents cumulative cases and D represents disease-induced deaths. The inclusion of $C$ and $D$ in the SEIR system does not alter its dynamics; it simply accounts for the exposed and deceased population. Data fitting is conducted using the logistic equation as the base function^21^.

6 $$\begin{array}{c}\left\{\begin{array}{c}\frac{dC}{dt}=\sigma E\\ \frac{dD}{dt}=f\gamma I\end{array}\right.\end{array}$$

### Procedure to construct the transmission rate

**Figure 1:**
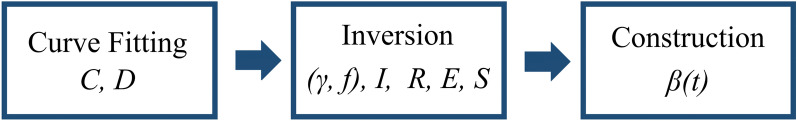
Procedure for estimating the time-dependent transmission rate.

We obtain regression functions by curve fitting the cumulative data of cases and deaths. Then, the values of $\gamma$ and $f$ are calculated using the linear least square method in Eqs. (8) and (9). All variables ${I}$,${R}$, ${E}$ and ${S}$ are determined, and the time-dependent transmission rate ($\beta \left(t\right)$) is constructed for various incubation periods, after which the time-dependent reproduction rate $\left({\text{R}}_{t}\right)$ is obtained. The overall procedure shown in Fig. 1 summarizes the algorithm to obtain $\beta \left(t\right)$.

$C$ and $D$ represent curve-fitted data of the cumulative cases and deaths. By solving the inverse problem consisting of Eqs. (1) and (6), we estimate $\gamma$, $f$, ${I}$, ${R}$, ${E}$ and ${S}$, and then $\beta \left(t\right)$.

### Curve fitting of $C$ and $D$

**Figure 2:**
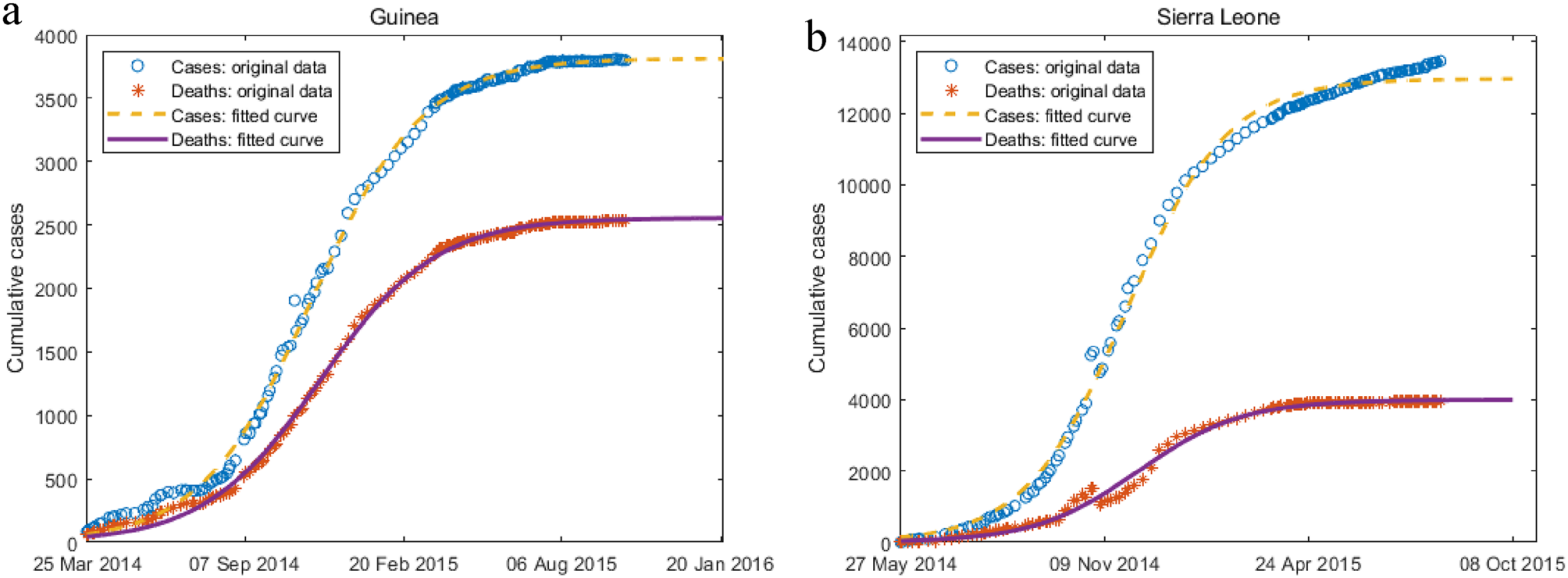
Curve fitting to the cumulative case and death data of Guinea (**a**) and Sierra Leone (**b**). The Levenberg–Marquardt method with 95% confidence bounds is used. R-squared values exceeding 0.948 are obtained for all cases (Guinea cases; 0.9999, deaths; 0.9999 and Sierra Leone cases; 0.9478, deaths; 0.9998). The curve fitting utilizes data points up to October 18, 2015 for Guinea and up to August 7, 2015 for Sierra Leone.

To estimate the values of parameters for the transmission dynamics Eq. (1), we fit the model to the cumulative data of cases $\left(C\right)$ and deaths $\left(D\right)$. Coefficients a, b, c and d are obtained from fitting the solutions of Eq. (7). We used a logistic function for regression with the Levenberg–Marquardt method^31,32^ in MATLAB^33^, as it is convenient and suitable for describing the proposed method and approximates very well. For Guinea, the R-squared values are 0.9999 for cases and 0.9999 for deaths in Fig. 2a. For Sierra Leone, the R-squared values are 0.9478 for cases and 0.9998 for deaths, as shown in Fig. 2b.

7 $$\begin{array}{c}\left\{\begin{array}{c}\frac{dC}{dt}=aC\left(1-bC\right)\\ \frac{dD}{dt}=cD\left(1-dD\right)\end{array}\right.\end{array}$$

### Estimation of removal rate $\left(\gamma \right)$ and fatality $\left(f\right)$

We assume that the total population size ${N}={S}+{E}+{I}+{R}+{D}$ in each country is constant and that the initial value of ${R}(0)=0.$

From Eq. (1), we have

8 $$\left\{ \begin{array}{c}\frac{1}{f\gamma }\frac{dD}{dt}=I \\ \frac{1}{f\gamma }\frac{{d}^{2}D}{d{t}^{2}}=\frac{dI}{dt}=\sigma E-\gamma I=\frac{dC}{dt}-\frac{1}{f}\frac{dD}{dt}\\ \frac{{d}^{2}D}{d{t}^{2}}= f\gamma \frac{dC}{dt}-\gamma \frac{dD}{dt}\end{array}\right.$$

It is rewritten as

9 $$\begin{array}{c}\left(\begin{array}{cc}\frac{dC}{dt}& -\end{array}\frac{dD}{dt}\right)\left(\begin{array}{c}f\gamma \\ \gamma \end{array}\right)=\frac{{d}^{2}D}{d{t}^{2}},\end{array}$$

The parameters $f$ and $\upgamma$ are estimated using the linear least square method or pseudoinverse.

For the simulation, we choose a data set from the beginning to various days during the Ebola outbreak^34^. The mean infectious time of Guinea is listed in Table 1. The mean infectious time is 10.46 days or 9.90 days if we use the dataset of 1–200 or 1–240 corresponding to October 16, 2015 and December 18, 2015, respectively. The simulation uses 10.46 days as the mean infectious time. For Sierra Leone, we take the mean infectious time as 10.14 days estimated by the data up to August 7, 2015. The fatality rate in Guinea is 65.86% and in Sierra Leone is 30.50%, and there is no significant change in either country from beginning to end.

**Table 1:** Mean infectious times and fatality for various sets of data.

Guinea				Sierra Leone			
Index	Date 2015	Mean infectious time	Fatality	Index	Date 2015	Mean infectious time	Fatality
120	5/24	17.79	0.6607	120	6/21	18.63	0.3037
140	7/3	14.25	0.6580	130	7/10	15.09	0.3036
160	7/31	12.42	0.6580	140	7/24	12.42	0.3041
180	9/4	11.26	0.6583	150	8/7	10.14	0.3050
200	10/16	10.46	0.6586	160	8/25	8.32	0.3062
220	11/17	10.39	0.6585	170	9/14	7.16	0.3078
240	12/18	9.90	0.6589	180	10/2	7.49	0.3098

The index represents the reported data number starting on March 25, 2014 for Guinea and May 27, 2014 for Sierra Leone. The mean infectious time $\left(\upgamma \right)$ for Guinea does not vary much after the 200th data point of October 16, 2015. The simulation uses 10.46 days as the mean infectious time. For Sierra Leone, the mean infectious time appears to decrease for a large data set but then starts to increase after index 170. Considering the mean infectious time in Guinea, it is picked at the index 150 where it is estimated by 10.14 days. The fatality rates in Guinea are between 65 and 66%, and those in Sierra Leone are between 30 and 31%.

### Construction of $\beta \left(t\right)$

The values of *S*, *E*, *I* and *R* are determined according to Eqs. (10)–(15). The parameters $f$ and $\upgamma$ are estimated using the linear least square method, as detailed in Table 1.

From Eq. (6),

10 $$\begin{array}{c}I(t)=\frac{1}{f\gamma }\frac{dD}{dt}\end{array}$$

Integrating $\frac{dR}{dt}$ in Eq. (1), we obtain

11 $$\begin{array}{c}R\left(t\right)=\left(1-f\right)\gamma\int_0^{t} {I }(t)dt,\end{array}$$

*E*(*t*) is calculated directly from Eq. (6)

12 $$\begin{array}{c}E(t)=\frac{1}{\sigma }\frac{dC}{dt}\end{array}$$

when $\upsigma$ is given.

Defining

$$\upphi \left(t\right)\stackrel{\scriptscriptstyle\text{def}}{=}\frac{\beta \left(t\right){SI}}{{N}}$$

we have

13 $$\begin{array}{c}\upphi \left(t\right)=\frac{dE}{dt}+\sigma E\end{array}$$

Integrating $\frac{dS}{dt}$ in Eq. (1), we obtain

14 $$\begin{array}{c}S\left(t\right)=S(0)-\int_0^{t}{\upphi \left(t\right)}dt\end{array}$$

The initial value

15 $$\begin{array}{c}S(0)=N-\left(E\left(0\right)+I\left(0\right)+R\left(0\right)+{D}\left(0\right)\right)\end{array}$$

Putting ${S}$ into $\upphi (t)$, we have

$$\beta \left(t\right) = \frac{{N}}{{{SI}}}\left( {\frac{dE}{dt}} + \sigma {E} \right)$$

### Transmission rate curves and the date of CP

**Figure 3:**
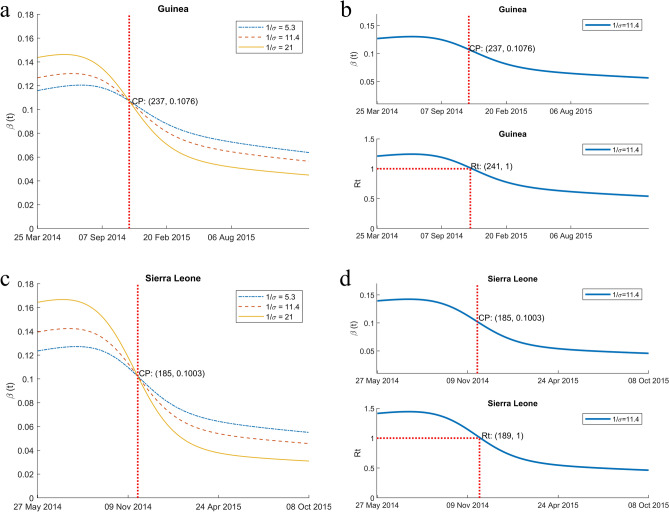
Transmission rate $(\beta \left(t\right))$ for various lengths of incubation period and reproduction rate ${\text{R}}_{t}$. (**a**, **c**) The transmission rate for various incubation periods ($1/\upsigma )$ are shown for Guinea and Sierra Leone. All transmission curves intersect at a single point (CP), which is independent of the incubation period. As the incubation period increases, the transmission rate exhibits a higher maximum value and decays more rapidly thereafter. CP (237, 0.1076) and CP (185, 0.1003) indicate that the transmission rate curves intersect at a common point on the 237th day with a transmission value of 0.1076 for Guinea and on the 185th day with a value of 0.1003 for Sierra Leone. (**b**, **d**) the plots depict *β*(*t*) and ${\text{R}}_{t}$ for incubation periods of 11.4 days for Guinea and 10.14 days for Sierra Leone. In both cases, CP appears approximately 4 days earlier than ${\text{R}}_{t}=1$.

The incubation period is defined as the interval between exposure to a pathogen and the initial occurrence of symptoms and signs^35^. The mean incubation period of Ebola virus disease ranges from 2 to 21 days depending on simulation methods, data and country^20,23,35^. For three different incubation periods, the time-dependent transmission rates are calculated. The first incubation period is 5.3 days according to the Ebola virus in Congo^23^. The second one is 11.4 days, based on data from the WHO Ebola Response Team^20^, and the third one is the maximum incubation days^35^. Figure 3a,c show the estimated transmission rates with various lengths of incubation periods ($1/\sigma$). The greater the value of the incubation period the greater the maximum value of the transmission rate and its decay rate. Additionally, one can also observe that the transmission rate curves intersect at a single point for three incubation periods, as shown in Theorem 1.

### Time comparison of CP and ${\text{R}}_{t}=1$ points

Table 2 shows the values of $E/S$, $\frac{\sigma E}{\upgamma I}$, and ${\text{R}}_{t}$ in Eq. (5) at CP. In both countries, ${\text{R}}_{t}>$ 1 at CP, indicating that CP is expected to appear earlier than ${\text{R}}_{t}=1$. For Guinea, ${\text{R}}_{t}=1$ appears on the 241st and CP on the 237th day from March 25, 2014. For Sierra Leone, it appears on the 189th day and CP on the 185th day from May 27, 2014 referring to Table four. The reference dates correspond to the time when ${\text{R}}_{t}=1$ in Fig. 3 are approximately nearby November 20, 2014 for Guinea and December 2, 2014 for Sierra Leone for the incubation period of 11.4 days (see Table 3). CP is very close to the date of ${\text{R}}_{t}=1$ and is independent of the incubation period. Therefore, CP can be an alternative indicator suggesting that the disease is very close to being under control, ${\text{R}}_{t}\approx 1$, even when the incubation period is unknown or estimated with high uncertainty.

**Table 2:** Information on $E/S$, $\frac{\sigma E}{\upgamma I}$, and ${\text{R}}_{t}=1$ at CP for Guinea and Sierra Leone.

	Guinea			Sierra Leone		
	$E/S$	$\frac{\sigma E}{\upgamma I}$	${\text{R}}_{t}$	$E/S$	$\frac{\sigma E}{\upgamma I}$	${\text{R}}_{t}$
At CP $\frac{{d}}{{{dt}}}\left( {E/S} \right)=0$	1.5885 × 10^−5^	$1.0267$	1.011	1.4759 × 10^−4^	$1.0351$	1.028

The time-dependent reproduction number $\left({\text{R}}_{t}\right)$ at CP is 1.011 for Guinea and 1.028 for Sierra Leone. Additionally, the other values of factors are $\frac{\sigma E}{\upgamma I} = 1.0267$, $\frac{1}{\left(1+E/S \right)}\approx 1$ for Guinea with a total population (*N*) of 11,745, and $\frac{\sigma E}{\upgamma I} = 1.0351$, $\frac{1}{\left(1+E/S \right)}\approx 1$ for Sierra Leone with $N=6092057$.

**Table 3:** The dates of CP and ${\text{R}}_{t}=1$.

Incidents	Guinea	Sierra Leone
	Date (2014)	Date (2014)
$dE/dt=0$	November 12–14	November 21–27
$\frac{{d}}{{{dt}}}\left( {E/S} \right)=0$::CP	November 15–16	November 27–28
$dE/dt+dI/dt=0$::${\text{R}}_{t}=1$	November 20–22	December 2–4
$dI/dt=0$	November 24–25	December 6–7

It indicates the dates when CP and ${\text{R}}_{t}=1$ appear between $dE/dt=0$ and $dI/dt=0$. The calendar date that corresponds to the cross point is November 15–16 for Guinea and November 27–28 for Sierra Leone. CP is reached approximately one week before the time-dependent reproduction rate decreases to one for $1/\upsigma =11.4$

Table 3 presents the time sequence in which four events occur, including CP, ${\text{R}}_{t}=1$, and the maximum values of *E*(*t*) and *I*(*t*). Using Eqs. (1) and (2), ${\text{R}}_{t}=1$ can be expressed as

16 $$\begin{array}{c}dE/dt+dI/dt=0\end{array}$$

${\text{R}}_{t}=1$ occurs only a few days after passing the time point of CP, as shown in Fig. 3b,d.

### Informations about the reported data and its corresponding dates

Table 4 shows the data indicated by the inflection point obtained from the regression curve in the reported data points. On November 12, the cumulative number of confirmed cases in Guinea jumped from 1878 to 1919 in two days, which corresponds to an index from 63 to 64 in Table 4. The number of inflection points from the regression is between 1894 and 1901. This means that $dE/dt=0$ occurs between November 12 and 14, 2014. In Sierra Leone, the inflection point from the regression is between 6329 and 6667. Hence, $dE/dt=0$ occurs between November 21 and 27, 2014. The calendar date that corresponds to CP is November 15–16 for Guinea and November 26–27 for Sierra Leone. The days of $dI/dt=0$ are November 24–25 and December 6–7. The time-dependent reproduction number is nearly 1 on November 20–22 for Guinea and December 2–4 for Sierra Leone. The time-dependent reproduction number (${\text{R}}_{t}$) at CP was 1.011 for Guinea and 1.028 for Sierra Leone, as shown in Table 2. The index refers to the order in which the number of cases was reported from March 25, 2014 for Guinea and May 27, 2014 for Sierra Leone.

**Table 4:** Reference dates for incidents.

Guinea		Sierra Leone	
Index	Cumulative cases	Index	Cumulative cases
	Days from March 25, 2014 (date)		Days from May 27, 2014 (date)
63	1878 cases	51	6190 cases
	233 days (November 12)		179 days (November 21)
64	1919 cases	52	6599 cases
	235 days (November 14)		184 days (November26)
65	1971 cases	53	7109 cases
	240 days (November 19)		186 days (November 28)
66	2047 cases	54	7312 cases
	242 days (November 21)		191 days (December3)
67	2134 cases	55	7897 cases
	247 days (November 26)		198 days (December 10)

Data points are not uniform. Data points are numbered from the beginning of March 25, 2014 for Guinea and May 27, 2014 for Sierra Leone. The index indicates the order in which the number of patients was reported.

### The ratio of $E/S$ for various $S(0)$

**Figure 4:**
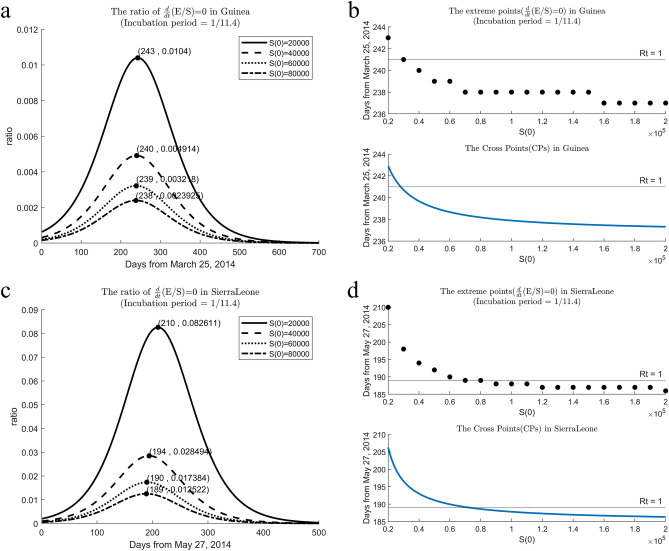
Tracing the extreme points of $E/S$ ratio and CPs for various cases of *S*(0) from 20,000 to 200,000. (**a**, **c**) Several $E/S$ ratios are estimated for $S(0)=\text{20,000}$ to 80,000 in increments of 20,000 units for Guinea and Sierra Leone. The maximum value appears near the 243rd day for $S(0)=\text{20,000}$ and the 238th day for $S(0) =\text{80,000}$ for Guinea. For Sierra Leone, it appears near the 210th day for $S(0)=\text{20,000}$ and the 189th day for $S(0) =\text{80,000}$ (**b**, **d**) (above): These graphs illustrate the dates when the maximum values of $E/S$ appear as the value of *S*(0) changes from 20,000 to 200,000. The days on which the $E/S$ values reach their maximum are March 25, 2014 for Guinea and May 27, 2014, for Sierra Leone. They converge to the 237th day for Guinea and the 185th day for Sierra Leone. (**b**, **d**) (below): The Cross Points are calculated from the two transmission rate curves. R_*t*_ = 1 is constant at 241st day for Guinea and 189th day for Sierra Leone for *S*(0).

Figure 4a,c illustrate the estimated $E/S$ ratios for four *S*(0) ​​ranging from 20,000 to 80,000 for Guinea and Sierra Leone. In Guinea, when *S*(0) is 20,000, the maximum value of $E/S$ occurs around the 243rd day. When *S*(0) is 60,000, it occurs around the 239th day, and for 80,000, it occurs approximately on the 238th day, as depicted in Fig. 4a. For Sierra Leone, when *S*(0) is 20,000, the maximum value of $E/S$ occurs around the 210th day. When *S*(0) is 80,000, the maximum value of $E/S$ occurs on the 189th day, as shown in Fig. 4c. The extreme values of the $E/S$ ratios are traced from the $E/S$ curves and CP in Fig. 4b,d. These extreme values are estimated pointwise from the $E/S$ ratio curves, while the Cross Points are calculated from two transmission rate curves. Since $dE/dt=0$ and $dI/dt=0$ can be estimated through Eqs. (10)–(12), the time of ${\text{R}}_{t}=1$ estimated from Eq. (16) is not affected by *S*(0). When *S*(0) is 20,000, ${\text{R}}_{t}=1$ appears before the maximum value of $E/S$. Until *S*(0) reaches 30,000 for Guinea and 60,000 for Sierra Leone, ${\text{R}}_{t}=1$ appears before the maximum value of $E/S$. For *S*(0) over 100,000, the maximum value of $E/S$ converges to the 237th day for Guinea and the 185th day for Sierra Leone, as shown in Fig. 4b,d. As *S*(0) increases beyond 80,000, the maximum value of $E/S$ appears before ${\text{R}}_{t}=1$ for both countries in Fig. 4b,d.

## Discussion

By solving the inverse problem, the time-dependent transmission rate is estimated using the cumulative data of Ebola outbreaks in Sierra Leone and Guinea between 2014 and 2016, and by rearranging the differential equation system of the SEIR model. The logistic equation (Eq. 7) fits very well to the data, as shown in Fig. 2, and it is very useful to explain the proposed algorithm. However, other statistical methods such as the adaptive Metropolis–Hastings (M–H) algorithm for the Bayesian Markov Chain Monte Carlo (MCMC) procedure can also be used^36^. After obtaining the appropriate regression function, the variables ($S$, $E$, $I$ and $R$) and parameters ($f,\gamma$) of the system can be found easily by the inverse method. Although the mean infectious time can be selected from other references^19,36^, the mean infectious time is estimated using cumulative data by its pseudoinverse (Eqs. 8, 9). If parameters ($f,\gamma$) are given, then Eqs. (1) and (2) are enough to estimate ${\text{R}}_{t}$, $E/S$, and CP.

We can track the *E*/*S* ratio by tracking the distance between the two transmission rate curves for the two incubation periods. If the distance between two transmission rate curves for the two incubation periods does not decrease, the infectious disease is continuously spreading, so quarantine measures must be further strengthened. At CP the difference becomes 0, it means that quarantine measures are being implemented appropriately.

The existence of CP is inherent in the SEIR model and, therefore, does not depend on data regression methods. From CP, it is observed that the transmission rate for a longer incubation period is lower than that for a shorter incubation period. The point at which $\frac{{d}}{{{dt}}}\left( {E/S} \right)=0$, or CP, represents the moment when the transmission rate shifts to a less transmissible rate for a longer incubation period.

The length of the incubation period varies greatly depending on the initial infection dose, the rate of pathogen replication, and the defense mechanisms within the host^37,38^. The possibility that the characteristics of the pathogen changed before and after CP cannot be ruled out. At CP or near ${\text{R}}_{t}=1$, the amount or pattern of replication of the pathogen in the host immune system may change. It is necessary to study changes in the incubation period depending on the characteristics of the pathogen within the host.

The accuracy of estimating the date of ${\text{R}}_{t}=1$ depends entirely on the precision of creating the regression function, which relies on different datasets, such as those up to August or September 2014. In this case study, the date of ${\text{R}}_{t}=1$ is estimated using the equation $dE/dt+dI/dt=0$, derived from Eqs. (1) and (2). The inflection points of cumulative data, such as when $dE/dt=0$ and $dI/dt=0$, appeared only a few days apart, as shown in Table 3. CP and ${\text{R}}_{t}=1$ occur between $dE/dt=0$ and $dI/dt=0$, which means that the occurrence times of CP and ${\text{R}}_{t}=1$ are very close. Although the timing of CP and ${\text{R}}_{t}=1$ depends on the value of $E/S$ in Eq. (5), they are very close to each other. The time-dependent reproduction number reaches one a few days after passing CP, indicating that the reproduction number is still greater than one at the time of CP. However, we can infer from CP that the epidemic will begin to decline within a few days. Thus, CP can be considered a new indicator that the epidemic is nearly under control. Moreover, since CP is not affected by the incubation period, it has the potential to serve as a criterion that can replace ${\text{R}}_{t}=1$ when there is uncertainty about the length of the incubation period.

The value of *S*(0) is a crucial factor that determines the temporal relationship between ${\text{R}}_{t}=1$ and CP. When *S*(0) is set to over 80,000, CP consistently appears earlier than ${\text{R}}_{t}=1$ for both countries, serving as a precautionary indicator that ${\text{R}}_{t}=1$ is imminent. However, assuming *S*(0) is small, such as 20,000, CP may appear after ${\text{R}}_{t}=1$, as shown in Fig. 4. In this case study, *S*(0) is assumed to be close to the total population, exceeding millions for both countries. Consequently, CP is expected to appear earlier than ${\text{R}}_{t}=1$, and this is also confirmed in the simulation.

## Conclusion

In solving the inverse problem of SEIR, we prove that transmission rate curves for various incubation periods intersect at a single point, denoted as CP (Cross Point), where $\frac{{d}}{{{dt}}}\left( {E/S} \right)=0$. The extreme value of the ratio $E/S$ occurs immediately before or immediately after ${\text{R}}_{t}=1$, depending on *S*(0). Therefore, in CP, ${\text{R}}_{t}=1$ is very close, so we can expect the epidemic to stabilize soon. The $E/S$ value can be estimated using incidence data or cumulative data in the inverse method, when the mean generation time and *S*(0) are given. Then the extreme value of $E/S$ can be traceable. However, tracing the CP is more convenient. By plotting transmission rate curves, *β*(*t*), for any two arbitrary incubation periods and tracking where they intersect, we can trace CP in time. Since CP is obtained using a random incubation period, accurate incubation period information is not required to find the extreme point of the ratio of $E/S$. Tracking $E/S$ ratio through other methods such as stochastic and artificial intelligence can be useful to predict and estimate the states of the epidemic. If *S*(*t*) is controlled by an effective vaccine or appropriate interventions, CP can be reached very quickly. This would be one way to get ${\text{R}}_{t}=1$ quickly.

## Data Availability

All data generated or analysed during this study are included in this published article. GitHub Reference: https://github.com/wuj2293/The-role-of-the-ES-ratio-in-the-SEIR-model.
